# Obstinate Vomiting of Pregnancy Treated with Ipecacuanha

**Published:** 1875-04

**Authors:** Meriweather Lewis

**Affiliations:** Lenoir’s, Tenn.


### OBSTINATE VOMITING OF PREGNANCY TREATED WITH IPECACUANHA.

BY MERIWEATHER LEWIS, M.D., OF LENOIR, TENN.

The following case is presented as illustrating a treatment comparatively new and untried:

Mrs. G. W. M., æt. 20, ten weeks advanced in her third pregnancy, was taken on the 5th of March, 1874, with vomiting. In her second pregnancy she had been reduced by a similar attack almost to death's door, in spite of active treatment.

Gradually growing worse, on the 10th of March her husband came for medicine, stating that everything swallowed was immediately vomited. Oxalate of cerium was ordered, grs. ij. three times a day. This was thrown up, dose after dose. On the 12th subcarbonate of bismuth was sent with no better result. Powdered ipecac. was then used in small doses given with essence of peppermint. Prompt relief followed. Doing without the ipecac., however, for a few days, brought on symptoms as severe as ever. Vomiting now occurred from twelve to twenty-four times a day.

Ipecac. was again ordered on the 18th, and on the 19th the patient was seen with a view of applying belladonna ointment to the cervix uteri. Finding, however, such marked improvement from the ipecac. sent on the preceding day, no local application was made, especially as the uterus was found normal in size and position corresponding with the period of gestation assigned. Continuing the treatment, a gradually increasing immunity from the attacks soon merged into complete relief, and by the 25th of

March the patient had resumed her household duties and pronounced herself entirely cured.

Of course, being a woman, she was not told what she was taking, otherwise failure, instead of success, would have been the result.

